# Alveolar ridge preservation reduces the need for ancillary bone augmentation in the context of implant therapy

**DOI:** 10.1002/JPER.22-0030

**Published:** 2022-04-29

**Authors:** Emilio Couso‐Queiruga, Cyrus J. Mansouri, Azeez A. Alade, Trishul V. Allareddy, Pablo Galindo‐Moreno, Gustavo Avila‐Ortiz

**Affiliations:** ^1^ Department of Periodontics University of Iowa College of Dentistry Iowa City IA USA; ^2^ Department of Epidemiology University of Iowa College of Public Health Iowa City IA USA; ^3^ Iowa Institute for Oral Health Research University of Iowa College of Dentistry Iowa City IA USA; ^4^ Department of Oral Pathology, Radiology and Medicine University of Iowa College of Dentistry Iowa City IA USA; ^5^ Department of Oral Surgery and Implant Dentistry, School of Dentistry University of Granada Granada Spain

**Keywords:** bone grafting, dental digital radiography, dental implants, periodontal atrophy, phenotype, tooth extraction

## Abstract

**Background:**

There is limited information on the need for bone augmentation in the context of delayed implant placement whether alveolar ridge preservation (ARP) is previously performed or not. The primary aim of this retrospective cohort study was to evaluate the efficacy of ARP therapy after tooth extraction compared with unassisted socket healing (USH) in reducing the need for ancillary bone augmentation before or at the time of implant placement.

**Methods:**

Adult subjects that underwent non‐molar single tooth extraction with or without simultaneous ARP therapy were included in this study. Cone beam computed tomography scans obtained before tooth extraction and after a variable healing period were used to record the baseline facial bone thickness and to virtually plan implant placement according to a standard method. A logistic regression model was used to evaluate the effect of facial alveolar bone thickness upon tooth extraction and baseline therapy (USH or ARP) on the need for additional bone augmentation, adjusting for several covariates (i.e., age, sex, baseline KMW, and tooth type).

**Results:**

One hundred and forty subjects that were equally distributed between both baseline therapy groups constituted the study population. Implant placement was deemed virtually feasible in all study sites. Simultaneous bone augmentation was considered necessary in 60% and 11.4% of the sites in the USH and ARP group, respectively. Most of these sites (64.2% in the USH group and 87.5% in the ARP group) exhibited a thin facial bone phenotype (<1 mm) at baseline. Logistic regression revealed that the odds of not needing ancillary bone augmentation were 17.8 times higher in sites that received ARP therapy. Furthermore, the need for additional bone augmentation was reduced 7.7 times for every 1 mm increase in facial bone thickness, regardless of baseline therapy.

**Conclusions:**

Based on a digital analysis, ARP therapy, compared with USH, and thick facial alveolar bone largely reduce the need for ancillary bone augmentation at the time of implant placement in non‐molar sites.

## 1 INTRODUCTION

Tooth extraction inevitably leads to a variable degree of alveolar ridge atrophy.^1^, ^2^, ^3^ Among other interceptive therapies,^4^, ^5^ contemporary evidence strongly supports the effectiveness of alveolar ridge preservation (ARP) therapy to prevent extensive alveolar ridge resorption after tooth extraction compared with unassisted socket healing (USH),^6^, ^7^ whether immediate implant placement is simultaneously performed or not.^8^

A systematic review and meta‐analysis evaluating the efficacy of different ARP modalities, concluded that ARP via socket grafting as compared with tooth extraction alone, prevents horizontal (1.99 mm), vertical mid‐buccal (1.72 mm) and vertical mid‐lingual (1.16 mm) bone resorption.^9^ Notably, it has been demonstrated that the efficacy of ARP therapy also depends on site‐specific factors, such as facial bone thickness at the time of tooth extraction.^10^, ^11^ Hence, adequate assessment and management of the extraction site can contribute to attenuate post‐extraction dimensional changes and preserve the architecture of the alveolar ridge. This is particularly critical in the anterior aesthetic zone when tooth replacement via implant therapy is planned.^12^

Dental implants have consolidated globally as the prime treatment option to replace missing teeth.^13^ The feasibility of implant placement in the ideal restorative position is related to a plethora of factors, such as anatomical characteristics of the site, patient‐related variables (e.g., range of mouth opening), macroscopic features of the implant fixture, prosthetic plan, and surgeon's preferences. Generally, with appropriate treatment planning and surgical execution, primary mechanical stability of the dental implant can be predictably achieved. However, in the presence of a deficient edentulous ridge, bone, and/or soft tissue augmentation procedures are often necessary before or at the time of implant placement in order to recreate an adequate biologic foundation,^14^, ^15^ which considerably increases the risk of morbidity, treatment expenses, and length of treatment time.^16^

Among available clinical studies on the topic of ARP therapy,^9^ there is limited information on the need for bone augmentation in the context of delayed implant therapy. The primary aim of this study was to evaluate the efficacy of ARP therapy following tooth extraction of non‐molar teeth compared with USH in reducing the need for ancillary bone augmentation procedures.

## 2 MATERIALS AND METHODS

This retrospective cohort study was conducted in the Department of Periodontics at The University of Iowa College of Dentistry and Dental Clinics according to the STROBE guidelines for cohort studies.^17^ Ethical approval was obtained from The University of Iowa Institutional Review Board (HawkIRB #202103604).

### 2.1 Sample size calculation

Based on data from a prior study,^10^ a sample size calculation was performed using the Open Epi sample size calculator. It was determined that additional bone augmentation at the time of implant placement was necessary in 13 of the 27 USH sites (48.1%) and three of the 26 ARP sites (11.5%). At 95% significance level and 80% power, the required sample size to detect a significant association between baseline therapy (USH versus ARP) and the need for additional augmentation ranged between 25 to 29 per group.  For the secondary predictor (i.e., facial bone thickness), the mean ± SD were 0.61 ± 0.31 mm and 0.98 ± 0.35 mm for USH and ARP, respectively. Using these values, the required sample size at 80% power and 5% type 1 error rate would be a minimum of 13 patients per group.

### 2.2 Eligibility criteria

Electronic health records of adult subjects that underwent non‐molar maxillary or mandibular single tooth extraction with or without simultaneous ARP via socket grafting and sealing between April 2013 and October 2021 were searched. The inclusion criteria were as follows: 1) ≥18 years of age; 2) ASA status I or II;^18^ 3) single maxillary or mandibular non‐molar tooth indicated for extraction and bound by natural, periodontally stable teeth; 4) cone beam computed tomography (CBCT) scans obtained and tooth extraction and after healing before implant placement planning; 5) alveolar bone integrity upon tooth extraction. The exclusion criteria were as follows: 1) mandibular incisors; 2) clinical attachment loss (AL) ≥2 mm affecting the study tooth; 3) current smokers or former smokers who quit within 6 months before tooth extraction; 4) uncontrolled diabetes mellitus, defined as HbA1c >7.0; 5) liver or kidney failure; 6) any active local or systemic acute infections; 7) any diseases or medications that may compromise normal wound healing; 8) currently receiving chemo‐or radiotherapy or a history of radiotherapy in head and neck area; 9) severe hematologic disorders; and 10) pregnancy or nursing mother.

### 2.3 Clinical procedures and digital data acquisition

All surgical procedures were performed by graduate residents. Before tooth extraction, a CBCT scan of the arch of interest was made. The field of view was approximately 6 cm at 0.3 mm voxel size and the exposure factor settings were fixed at 120 kVp, 18.66 mAs and 8.9 seconds of acquisition time.¶ Mid‐facial keratinized mucosa width (KMW) before tooth extraction was obtained from the patient's electronic record. All tooth extraction interventions were performed under local anesthesia in a flapless, minimally invasive fashion. Alveolar sockets were gently curetted and inspected upon extraction. In the USH group no further treatment was provided. In the ARP group, socket grafting, and sealing were performed. Sockets were filled up to the level of the alveolar crest, using either a particulate allograft composed of a mixture of 70% FDBA and 30% DFDBA# or with collagenated anorganic bovine bone mineral.*

The sockets that received the allograft mixture were sealed with a dense polytetrafluoroethylene (dPTFE) barrier membrane,† which was stabilized with a cross‐mattress dPTFE suture placed over the socket.‡ In the sites that received the xenograft material, the socket orifice was covered with a collagen matrix§ that was secured with four to six simple interrupted sutures.** All subjects received detailed verbal and written postoperative instructions, as well as prescriptions for anti‐inflammatory and pain reliever medications (ibuprofen 600 mg three times a day for 3 to 5 days), unless contraindicated for individual medical reasons. Patients in the ARP group were prescribed an antibiotic (amoxicillin [500 mg] every 8 hours for 7 days or, in case of penicillin allergy, clindamycin [300 mg] every 6 hours for 7 days). Sutures were removed after ≈ 2 weeks. After a variable healing period of 10 to 36 weeks, a second CBCT scan was acquired following the same with the same acquisition parameters described at baseline.

### 2.4 Data collection

Digital Imaging and Communication in Medicine (DICOM) files obtained from the CBCT scans were assessed by two independent masked examiners using a software package.†† To ensure data quality, one independent masked examiner (CM) performed linear measurements to determine facial bone thickness at baseline in ten random patients. Another independent masked examiner (ECQ) virtually planned implant placement in 10 random patients. An inter‐class correlation coefficient of at least 0.9 was achieved, after which data collection ensued.

**FIGURE 1:**
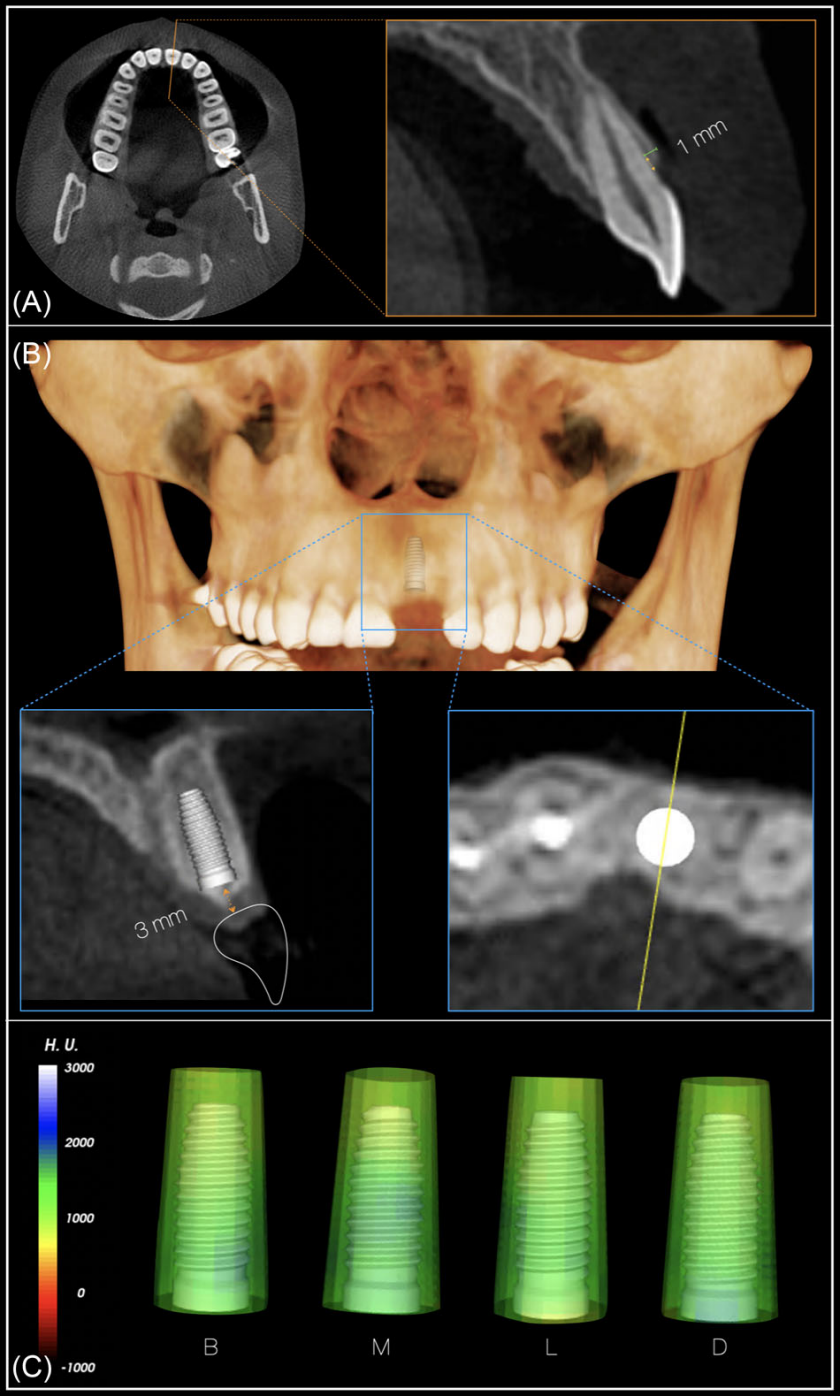
Visual depiction of the methodology followed to determine buccal bone thickness before tooth extraction (**A**). Virtual implant placement in a prosthetically and anatomical favorable location (**B**). Circumferential assessment of bone thickness around the implant (**C**)

To standardize the linear measurements of facial alveolar bone thickness, a sagittal section at the middle of each tooth before extraction was obtained using the baseline CBCT data, as described in a previous publication.^19^ A horizontal line, perpendicular to the long axis of the axial root plane, was drawn to intersect the facial‐most point of the facial bone and the tooth surface to subsequently measure the alveolar bone thickness at 1 mm apical to the crest, as shown in Figure 1A.

In the second DICOM file, a digital crown from the software digital library was imported and modified according to the characteristics of the edentulous site and adjacent teeth. A generic bone level implant with a diameter of 4.0 mm and a length of 9.0 mm was virtually placed in all sites, except for maxillary lateral incisors, where an implant with a 3.5 mm diameter and 9.0 mm of length was used. Implants were placed in alignment with the long axis of the ridge and 3 mm apical to the mid‐facial zenith of the digital crown to respect a minimum of 3 mm of supracrestal tissue height.^20^ Implant placement was considered feasible if achieving primary mechanical stability of the dental implant was likely based on the anatomical characteristics of the supporting bone relative to the virtual implant fixture. Additional bone augmentation was deemed necessary if a minimum of 1 mm of circumferential bone support was not observed around the whole implant fixture, as shown in Figure 1B.

### 2.5 Statistical analyses

The primary predictor variable (baseline therapy: ARP or USH) and the dependent variable (need for bone augmentation: yes or no) were recorded as binary. Covariates included age, sex, baseline KMW, and tooth type. All of them were analyzed as categorical variables, except age, which was analyzed as a continuous variable. A logistic regression model was used to evaluate the relationship between baseline therapy and the need for ancillary bone augmentation, adjusting for the covariates. Additionally, the association between facial bone thickness (secondary predictor and analyzed as a continuous variable) and the need for bone augmentation was evaluated. Bone phenotype measured in the DICOM files at baseline was dichotomized (facial bone thickness < 1 mm as thin and ≥1 mm as thick) according to available evidence^1^, ^10^ and used in the logistic regression model as previously explained. The assumptions of logistic regression were evaluated using the Cook's distance and standardized residuals to check for influential observations and variance inflation factors that would be indicative of multicollinearity. All data analyses were performed using a specific software package.‡‡

## 3 RESULTS

### 3.1 Population

A total of 140 patients constituted the study sample, of which 70 (35 females and 35 males) were in the USH group and 70 (40 females and 30 males) in the ARP group. Among them, 26 subjects underwent ARP therapy consisting of a combination of particulate bone allograft and dPTFE barrier membrane for socket sealing, whereas 44 subjects received a combination of collagenated anorganic bovine bone mineral and a porcine collagen matrix to seal the extraction socket. Mean age of the study population was 58.2 ± 14.8 years (USH, 59 ± 15.2; ARP, 57.4 ± 14.4).

### 3.2 Baseline information

Mean values were similar among groups for baseline facial bone thickness (USH, 1.15 ± 0.57 mm; ARP, 1.18 ± 0.62 mm) and facial KMW (USH, 4.37 ± 1.3 mm; ARP, 4.36 ± 1.56 mm). Fifty‐three patients presented a thin bone phenotype (USH, n = 30; ARP, n = 23) with a mean thickness of 0.59 ± 0.25 mm (USH, 0.61 ± 0.24 mm; ARP, 0.55 ± 0.26 mm) and with a mean KMW of 4.0 ± 1.3 mm (USH, 4.3 ± 1.24 mm; ARP, 3.61 ± 1.27 mm). On the other hand, 87 patients presented a thick bone phenotype (USH, n = 40; ARP, n = 47) with a mean facial bone thickness of 1.51 ± 0.45 mm (USH, 1.55 ± 0.38; ARP, 1.48 ± 0.5) and with a mean KMW of 4.57 ± 1.48 mm (USH, 4.4 ± 1.35 mm; ARP, 4.72 ± 1.57 mm). Tooth type distribution was similar in both groups, including a total of 13 maxillary central incisors (USH, n = 5; ARP, n = 8), 26 maxillary lateral incisors (USH, n = 11; ARP, n = 15), 7 maxillary canines (USH, n = 3; ARP, n = 4), 40 maxillary first premolars (USH, n = 28; ARP, n = 12), 41 maxillary second premolars (USH, n = 19; ARP, n = 22), 2 mandibular canines (USH, n = 0; ARP, n = 2), 3 mandibular first premolars (USH, n = 0; ARP, n = 3), and 8 mandibular seconds premolars (USH, n = 4; ARP, n = 4). Reasons for extraction included tooth cracks (n = 77), extensive caries (n = 44), root resorption (n = 5), restorative reasons (n = 10), and endodontic complications (n = 4).

### 3.3 Outcomes

**FIGURE 2:**
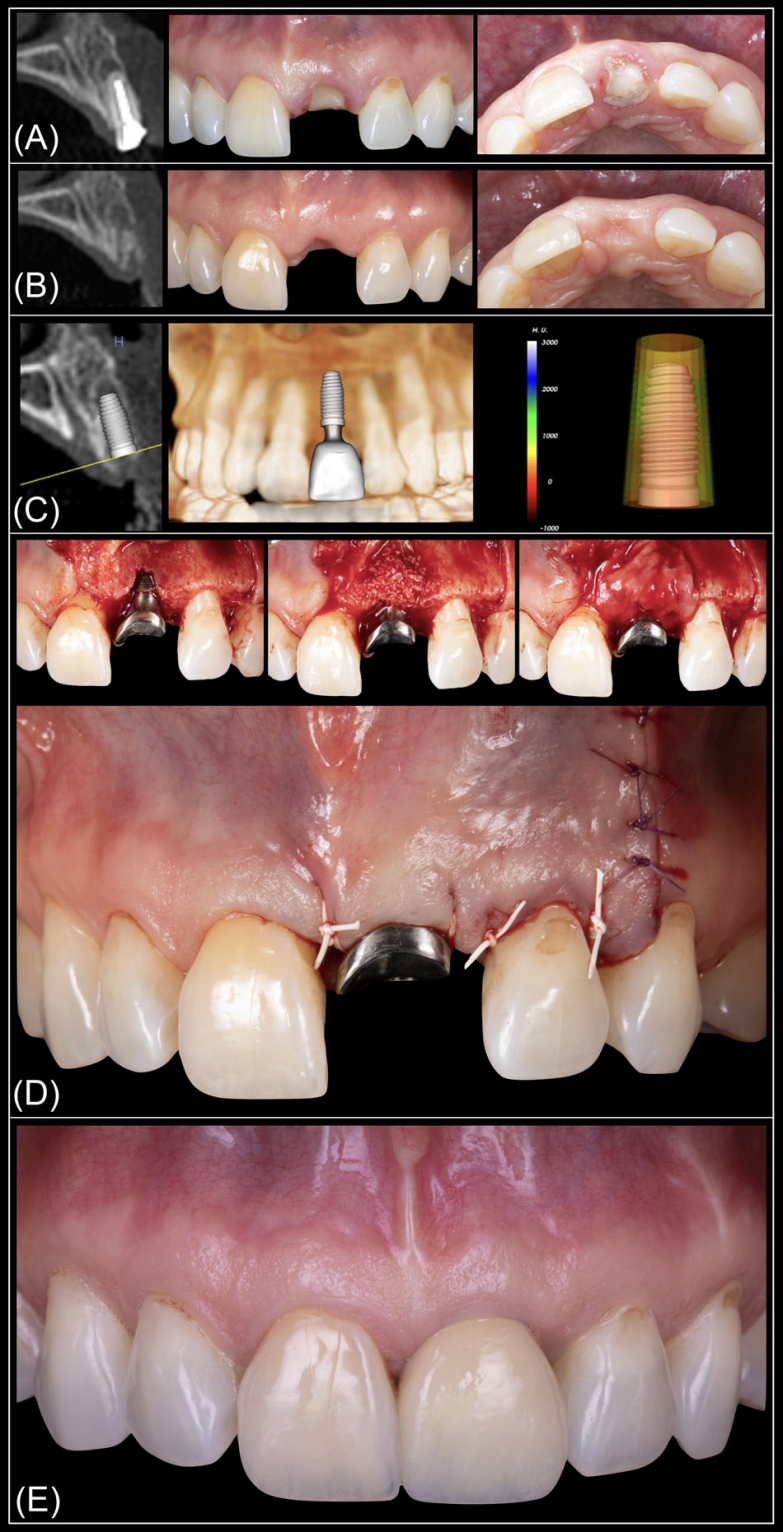
Representative case of a site presenting a thin bone phenotype. Radiographic and clinical aspect at baseline (**A**) and after ≈ 3 months of healing (**B**). Additional bone augmentation was deemed necessary upon virtual implant placement (**C**) and confirmed at the time of implant placement (**D**). Frontal intraoral view after delivery of the final restoration (**E**). Case restored by Dr. Christopher Barwacz, Department of Family Dentistry, University of Iowa College of Dentistry

**FIGURE 3:**
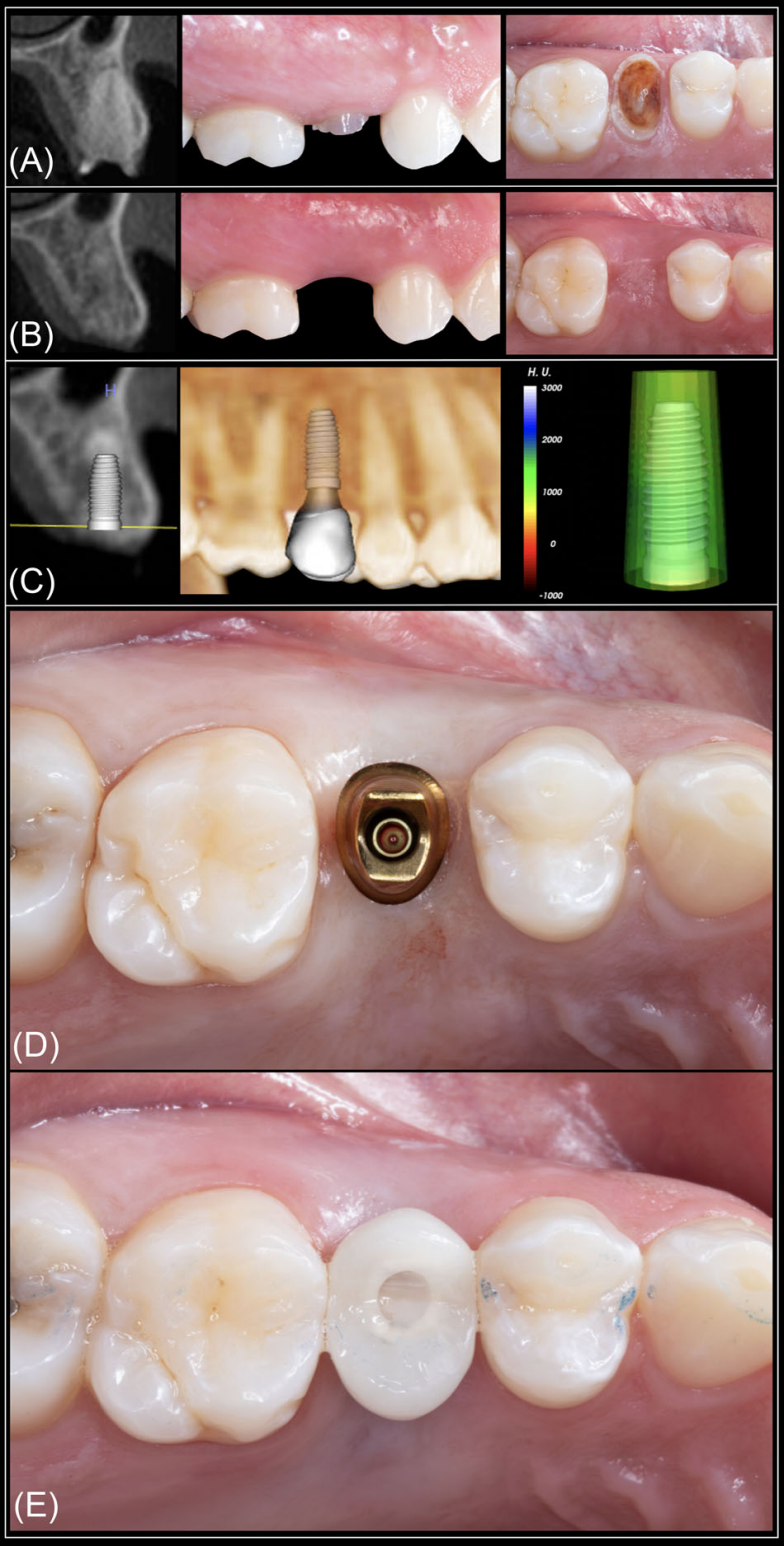
Representative case of a site presenting a thick bone phenotype. Radiographic and clinical aspect at baseline (**A**) and after ≈ 6 months of healing (**B**). After virtual implant placement, it was determined with a high degree of certainty that no additional bone augmentation would be necessary (**C**). Static computer‐aided implant placement was planned and executed (**D**). Primary stability was achieved, and a provisional custom‐made implant‐supported restoration was delivered (**E**). Case restored by Dr. Christopher Barwacz, Department of Family Dentistry, University of Iowa College of Dentistry

**FIGURE 4:**
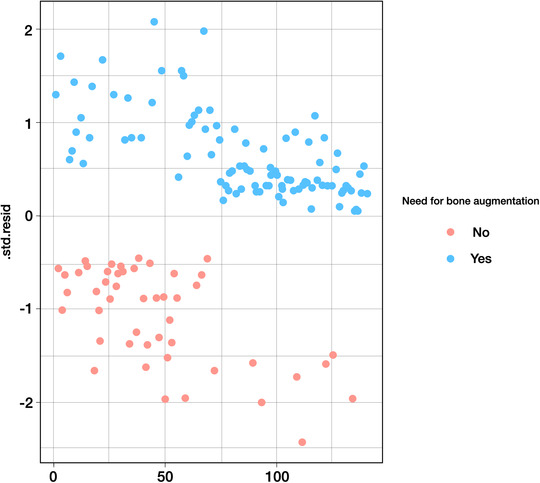
Plausibility of the assumptions made in the logistic regression model was evaluated using the standardized residuals. The results of this assessment ruled out the presence of influential values (extreme values or outliers) which may have driven the observed associations

Implant placement was deemed virtually feasible in all study sites, indicating that no need for site development before implant placement would be required. In the USH group, implant placement with simultaneous ancillary bone augmentation was deemed as necessary in 42 of 70 sites (60%). Twenty‐seven of these 42 sites (64.2%) presented a thin bone phenotype at baseline. Twenty‐seven of 30 sites (90%) presenting a thin bone phenotype at baseline required additional bone augmentation procedures. In the ARP group, it was determined that eight of the 70 sites (11.4%) would require ancillary bone augmentation at the time of implant placement. Seven of these eight sites (87.5%) had a thin periodontal bone phenotype at baseline. Seven of 23 sites (30.4%) presenting a thin bone phenotype at baseline required additional bone augmentation procedures. The results of the logistic regression analysis, adjusting for age, sex, KMW, and facial bone thickness at baseline, are displayed in Table 1. This analysis revealed that the odds of not needing ancillary bone augmentation among sites in the ARP group was 17.8 times higher (95% CI, 6.6‒55.9) compared with the USH group. Regarding the relationship between facial bone thickness at baseline and need for ancillary bone augmentation placement, this analysis revealed that the need for additional bone augmentation was reduced 7.7 times for every 1 mm increase in facial bone thickness (95% CI, 3.2‒21.7). Interestingly, the association between facial bone thickness at baseline and need for bone augmentation adjusting for age, sex, and KMW did not differ in function of baseline therapy. Representative cases of sites presenting a thin and thick bone phenotype at baseline are depicted in Figures 2 and 3, respectively. No statistically significant association was observed between age, sex, baseline KMW, or tooth type and the need for bone augmentation. However, the association of need for bone augmentation with baseline therapy and facial bone thickness remained significant after adjusting for tooth type, as shown in Table 2. Examination of the standardized residuals did not suggest the presence of any influential observation or outliers (Figure 4). Furthermore, the correlation between the different combinations of the predictors was examined to check for multicollinearity. The highest variance inflation factor observed was <1.5, making multicollinearity very unlikely (see Supplementary Table 1 in online *Journal of Periodontology*).

**TABLE 1:** Logistic regression model showing the association between the need for ancillary bone augmentation at the time of implant placement and ARP therapy, age, sex, facial bone thickness, and facial keratinized mucosa width

	Odds	95% CI	*P* value
ARP treatment	17.80	6.63‒55.98	9.34E‐08*
Age	1.02	0.99‒1.06	0.11561
Sex	0.71	0.28‒1.74	0.44928
Buccal bone thickness	7.77	3.17‒21.70	2.59E‐05*
Keratinized tissue width	1.12	0.79‒1.59	0.52193

CI, confidence interval.

* Indicates statistical significance (*P* <0.05).

**TABLE 2:** Variance tests for the association between all the predictors and the need for ancillary bone augmentation at the time of implant placement

	Df	Deviance	Residual Df	Residual deviance	*P* value
Treatment	1	34.655	138	145.36	3.94E‐09*
Age	1	1.04	137	144.32	0.3079
Sex	1	0.044	136	144.28	0.8344
Buccal bone thickness	1	23.427	135	120.85	1.30E‐06*
Keratinized tissue width	1	0.416	134	120.43	0.519
Tooth types	7	10.216	127	110.22	0.1767

Df, degree of freedom.

* Indicates statistical significance (*P* <0.05).

## 4 DISCUSSION

This retrospective study was aimed at evaluating the efficacy of ARP therapy following tooth extraction, as compared with USH, in reducing the need for ancillary bone augmentation in the context of delayed implant therapy. The effect of age, sex, facial bone thickness and KMW on this relationship was explored. It must be noted that all study sites presented <2 mm of clinical AL and the alveolar sockets after tooth extraction were intact or minimally damaged. The exclusion of sites presenting severe bone damage after tooth extraction was made to homogenize the sample and eliminate the influence of major anatomic variability on the outcomes.^21^

Implant placement was deemed feasible in all 140 sites included in this study. One of the main findings was that ARP therapy was strongly associated with a reduced need for simultaneous bone augmentation at the time of implant placement as compared with USH group (88.6% versus 40%, respectively). Furthermore, the odds of not needing ancillary bone augmentation were 17.8 times higher in sites that received ARP therapy. Hence, the differences observed between groups can be largely explained by the treatment provided after tooth extraction. It is well known that ARP therapy attenuates post‐extraction dimensional changes of the alveolar ridge, particularly on the facio‐coronal aspect.^9^, ^10^ On the contrary, a more accentuated phenomenon of progressive alveolar ridge atrophy should be expected in sites that are left to heal after tooth extraction with no further intervention.^22^, ^23^ A recent systematic review that analyzed clinical evidence pertaining to post‐extraction dimensional changes after USH reported that non‐molar sites are associated with an increased need for ancillary bone augmentation to facilitate implant therapy (69.7%) compared with molar sites (45.9%).^3^ The observations are in alignment with the findings of the present study, where additional bone grafting augmentation procedures were deemed to be necessary in 60% of sites that did not received ARP therapy.

Another relevant finding of our study was that, independently of baseline therapy, age, sex, and KMW, the odds of needing ancillary bone augmentation were reduced 7.7 times for every 1 mm increase in facial alveolar bone thickness at baseline. Remarkably, 64.2% and 87.5% of sites that would require additional bone augmentation in the USH and ARP groups, respectively, exhibited a thin facial bone phenotype. To the best of our knowledge, this is the first study demonstrating that facial alveolar bone thickness influences the need for bone augmentation at the time of implant placement, whether ARP is performed or not. This finding is in accordance with the results of previous clinical studies, where facial bone thickness was identified as a strong predictor of the extent and magnitude of alveolar bone resorption after tooth extraction.^1^, ^10^, ^11^ This information highlights the critical importance of adequately managing fresh extraction sites presenting <1 mm of facial alveolar bone thickness. Interestingly, tooth type did not influence the outcomes in either group, which is likely related to the inclusion of non‐molar teeth with similar anatomical root features.^24^

Digital workflows for tooth replacement therapy focused on optimal three‐dimensional implant placement based on anatomical and prosthetic parameters offer numerous advantages to both optimize the outcomes of implant therapy and to expand the latitude of research methodologies.^25^, ^26^ Implant dimensions used for the virtual implant placement component of this study were predetermined on the basis of relevant clinical, prosthetic, and anatomical factors, in alignment with contemporary standards of care. Implants were placed to support a screw‐retained prosthesis. The effect of prosthetic solutions that may compensate up several degrees of axial discrepancy^27^, ^28^ and, subsequently, reduce the need for bone augmentation was not assessed. After virtual implant placement was completed, additional bone augmentation was deemed necessary when a minimum of 1 mm of circumferential bone support was not observed around the whole implant fixture. This decision was made in congruence with available preclinical and clinical evidence and the typical anatomical characteristics of the periodontium in non‐molar sites.^1^, ^2^, ^29^, ^30^ However, it must be acknowledged that, currently, there is a lack of consensus regarding a minimum threshold of peri‐implant bone thickness that would be necessary to achieve predictable esthetics, health, and/or peri‐implant tissue stability.^20^ Interestingly, a clinical study demonstrated that implants associated with small (≤5 mm) non‐contained facial bone dehiscence at the time of placement that did not receive any augmentation therapy showed high survival rates and healthy peri‐implant tissues at 7.5 years, with outcomes comparable to sites that underwent bone augmentation.^31^ Furthermore, other studies have shown that peri‐implant soft tissue augmentation with an autogenous subepithelial connective tissue graft and peri‐implant bone augmentation via guided bone regeneration are equally effective in reestablishing an adequate facial contour around single implants,^32^, ^33^ and rendered similar outcomes in terms of apical migration of the mucosal margin over time.^34^ The findings from these studies certainly challenges the need for circumferential peri‐implant bone support to obtain optimal outcomes. Nonetheless, some investigators have observed more favorable implant therapy outcomes in the presence of a thick peri‐implant bone phenotype as opposed to sites exhibiting thin bone.^35^, ^36^

This study has several limitations. First, only non‐molar sites were included. Therefore, the findings of this study should be interpreted with caution when making clinical decisions in molar sites.^24^, ^37^ Second, the effect of other local phenotypic characteristics (i.e., palatal bone thickness or gingival thickness) was not evaluated. This was a deliberate decision considering the negligible effect that these variables have on post‐extraction alveolar ridge dynamic, as reported in previous studies.^10^, ^38^ Third, while it is well documented that most dimensional changes affecting the alveolar ridge typically occur within the first 4 to 6 weeks after tooth extraction,^3^, ^39^ the wide range of healing time (10 to 36 weeks) may have slightly influenced the observed outcomes. Fourth, as it was previously alluded to, implants were virtually placed according to a restorative plan compatible with a screw‐retained implant prosthesis, disregarding prosthetic options to compensate discrepancies from the ideal implant axis, such as angulated screw access channels, which may reduce or avoid the need for ancillary bone augmentation. Although digital evaluation could be considered a limitation, a previously published study concluded that there is no pattern of underestimation or overestimation when digital measurements are compared with direct measurements.^40^ Fifth, the retrospective nature of this study does not allow to establish causal relationships between confounding factors and to determine the reasons that guided the clinical decision‐making process (i.e., ARP versus USH) in the cases included in our cohort.

## 5 CONCLUSIONS

Based on implant software modeling, ARP therapy reduces substantially the projected need for ancillary bone augmentation at the time of implant placement compared with USH. Facial bone thickness is strongly associated with the need for alveolar bone augmentation independently of the treatment provided. The thicker the facial alveolar bone at baseline, the higher the chances of implant placement in a prosthetically acceptable position without the need for ancillary bone augmentation. This information can be utilized in daily clinical practice to make evidence‐based decisions for adequate management of the extraction site in non‐molar areas, particularly when tooth replacement via delayed implant therapy is planned.

## CONFLICT OF INTEREST

The authors report no conflicts of interest related to this study.

## AUTHOR CONTRIBUTIONS

Dr. Couso‐Queiruga and Prof. Avila‐Ortiz conceived the idea. Drs. Couso‐Queiruga, Mansouri, and Alade and Prof. Allareddy contributed to the study design, data acquisition, and analysis. Dr. Couso‐Queiruga and Prof. Avila‐Ortiz led the writing. Drs. Mansouri and Alade and Profs. Allareddy and Galindo‐Moreno critically revised the manuscript. All authors gave final approval and agreed to be accountable for all aspects of the scientific work.

## Supporting information

Supporting Information

## Footnotes

¶ i‐CAT Next Generation, Imaging Sciences International, Hatfield, PA

# enCore, Osteogenics Biomedical, Lubbock, TX

* Bio‐Oss Collagen, Geistlich Pharma, Wolhusen, Switzerland

† Cytoplast TXT‐200 Singles, Osteogenics Biomedical, Lubbock, TX

‡ Cytoplast 5‐0 suture, Osteogenics Biomedical, Lubbock, TX

§ Mucograft Seal, Geistlich Pharma, Wolhusen, Switzerland

** Resolon 6‐0, Resorba Medical, Nürnberg, Germany

†† InVivo v.5.3, Anatomage, Santa Clara, CA

‡‡ R studio version 4.0.3.0, Boston, MA
