# Supplementary material for: Alveolar ridge preservation reduces the need for ancillary bone augmentation in the context of implant therapy
Source: J Periodontol. 2022 Apr 29;93(6):847–56. doi: 10.1002/JPER.22-0030 (PMC9322559; doi:10.1002/JPER.22-0030)
Supplement: Supplementary file 1 — Supporting Information [file JPER-93-847-s001.docx]

| Variable | VIF |
| --- | --- |
| Treatment group | 1.24 |
| Gender | 1.03 |
| Keratinized tissue width | 1.09 |
| Age | 1.10 |
| Buccal bone thickness | 1.22 |

**Supplementary Table 1.**  Assessment of multicollinearity between the independent variables in the model by estimating the variance inflation factor (VIF). According to these results, there was no collinearity (A VIF value that exceeds 5 is indicative of collinearity among the predictor variables).
